# Editorial: Immune Regulation of Metabolic Homeostasis

**DOI:** 10.3389/fendo.2022.929460

**Published:** 2022-05-31

**Authors:** Bruno Guigas, Tony Jourdan, Rinke Stienstra

**Affiliations:** ^1^ Department of Parasitology, Leiden University Medical Center, Leiden, Netherlands; ^2^ INSERM Lipids, Nutrition, Cancer (LNC) UMR1231, Team PADYS, University of Burgundy and Franche-Comté, Dijon, France; ^3^ Department of Internal Medicine, Radboud University Medical Center, Nijmegen, Netherlands; ^4^ Nutrition, Metabolism and Genomics Group, Division of Human Nutrition and Health, Wageningen University, Wageningen, Netherlands

**Keywords:** immunometabolism, obesity, type 2 diabetes, NASH, macrophages, dendritic cells, T cells, metaflammation

The worldwide prevalence of obesity, non-alcoholic fatty liver disease (NAFLD) and type 2 diabetes is reaching epidemic proportions, not only in Western societies but also in developing countries (1, 2). Among various pathophysiological underlying mechanisms, the obesity-associated chronic low-grade inflammation, also called meta-inflammation or metaflammation, contributes to the development of insulin resistance and dysregulated glucose/lipid metabolism, ultimately leading to type 2 diabetes, non-alcoholic steatohepatitis (NASH), and associated cardiovascular diseases such as atherosclerosis (3–5). During the last decade, landmark studies in the emerging field of immunometabolism have highlighted the central role played by the immune system in the regulation of metabolic homeostasis in both rodent and humans. Indeed, a growing repertoire of innate and adaptive immune cells have been reported to populate metabolic organs, including adipose tissue, liver, pancreas, skeletal muscle, intestine and some brain areas, and to contribute to tissue-specific maintenance of insulin sensitivity and/or biological functions through complex but yet incompletely understood crosstalk with metabolic cells. Furthermore, the tissue microenvironment (nutrients, metabolites, oxygen,…) also play an important role in the regulation of intrinsic metabolism and functions of resident and/or recruited immune cell subsets within metabolic organs (6, 7). This finely-tuned and dynamic homeostatic system is altered in the context of obesity and metabolic disorders, bringing up the idea that modulation of immune environment in metabolic organs could constitute an attractive new therapeutic approach for alleviating metaflammation and cardiometabolic diseases (8, 9).

The present Research Topic provides a collection of high-quality manuscripts focused on different aspects of the immune regulation of metabolic homeostasis, notably in the context of obesity and type 2 diabetes. This issue comprises twelve manuscripts, including 6 original research articles and 6 reviews, that could be divided in 3 interrelated parts.

The first section is mainly dealing with the role of tissue-resident immune cells, especially from the myeloid lineage, in the pathophysiological regulation of nutrient metabolism and insulin sensitivity in various metabolic organs. As the first one of a series of state-of-the-art reviews, Khan et al. summarize the current knowledge on the changes in immune cell composition in visceral adipose tissue during obesity and aging, and discuss the respective contribution of innate and adaptive immune cell types to tissue dysfunction. In the light of the recent development of single cell technologies that have revealed heterogenous immune cell populations within metabolic organs (10–15), Remmerie et al. provide an overview of the distinct macrophage subsets identified in both adipose tissue and liver, and their putative functions during the development of obesity and fatty liver disease. In an extensive review, Orliaguet et al. describe the main underlying molecular mechanisms by which tissue-resident macrophages can be reprogrammed by obesogenic micro-environment in pancreatic islets, adipose tissue and the liver, and how changes in their polarization state can affect organ functions and systemic insulin sensitivity. The role of micro-environment in shaping myeloid cell functions is expanded to dendritic cells by Brombacher and Everts who dissect how changes in local nutrients or oxygen tension could modulate intrinsic metabolic pathways and affect cell differentiation and immunogenicity in pathological conditions like cancer and type 2 diabetes. Focusing on the interaction between adipocytes and invariant natural killer T (iNKT) cells within adipose tissue, van Eijkeren et al. highlight the putative role played by local adipocyte-derived lipids in shaping tissue-resident iNKT cell functions. In an original study, Surendar et al. investigate the role of adipose tissue and liver macrophages in CD8^+^ T cell functions during weight loss in diet-induced obese mice. Finally, in the last article of this section, Koc et al. report some differences in the transcriptional metabolic signatures of CD14^+^ and CD14^-^ peripheral blood mononuclear cells isolated from control subjects and age/weight-matched first-degree relatives of patients with type 2 diabetes.

In a second part, three original manuscripts describe various interventional approaches leading to improvements of metaflammation, insulin sensitivity and/or glucose homeostasis in mouse models of obesity and type 2 diabetes. Parasitic helminths are known to be master regulators of host immune responses through secretion of a portfolio of unique immunomodulatory molecules (16, 17). Interestingly, Khudhair et al. show that gastrointestinal helminth infection with the nematode *Nippostrongylus brasiliensis* induces a potent type 2 immune response in metabolic organs, and reduces body weight, systemic inflammation and hyperglycemia in high-fat diet-fed mice, a beneficial effect associated with changes in gut microbiota composition and increased fecal short chain fatty acid levels. In the second study, Romero-Zerbo et al. report the beneficial effects of the atypical cannabinoid Abn-CBD, a synthetic cannabidiol derivative with immunomodulatory properties, on both systemic and tissue-specific inflammatory parameters in diet-induced obese mice. Finally, Permyakova et al. describe how AN1284, a novel indoline derivative with antioxidant and anti-inflammatory activities, improves whole-body insulin sensitivity, reduces hepatic and renal inflammation and preserves kidney functions in BSK-*db*/*db* mice, a genetic model of diabetic obesity.

Finally, the last section of this Research Topic comprises two review articles focused on bone homeostasis. Benova and Tencerova summarize the current knowledge on the impact of obesity and lifestyle interventions on bone marrow microenvironment and subsequent remodelling of both hematopoietic and mesenchymal stem cells. In a mini-review, Cooney et al. discuss the role played by gut microbiota in the regulation of bone mass, notably through modulation of osteoclastogenesis by local immune cells, and how probiotics may influence this gut-bone axis.

Overall, the studies presented in this Research Topic highlight the central role played by the immune system in the (dys)regulation of many aspects of metabolic homeostasis, at both organ and systemic levels, especially during the development of obesity, type 2 diabetes and associated inflammatory-driven comorbidities. The expanding field of immunometabolism is expected to i) contribute to a better understanding of the role and functions of a growing numbers of tissue-specific innate and adaptive immune cell subsets, and ii) pave the way to the development of new immunomodulatory therapeutic strategies for the treatment of cardiometabolic diseases.

## Author Contributions

All authors listed have made a substantial, direct, and intellectual contribution to the work and approved it for publication.

## Conflict of Interest

The authors declare that the research was conducted in the absence of any commercial or financial relationships that could be construed as a potential conflict of interest.

## Publisher’s Note

All claims expressed in this article are solely those of the authors and do not necessarily represent those of their affiliated organizations, or those of the publisher, the editors and the reviewers. Any product that may be evaluated in this article, or claim that may be made by its manufacturer, is not guaranteed or endorsed by the publisher.

## References

[1] WHO. Obesity and Overweight. Key Facts. Available at: https://www.who.int/news-room/fact-sheets/detail/obesity-and-overweight.

[2] Paik JM Golabi P Younossi Y Mishra A Younossi ZM. Changes in the Global Burden of Chronic Liver Diseases From 2012 to 2017: The Growing Impact of NAFLD. Hepatology (2020) 72:1605–16. doi: 10.1002/hep.31173 32043613

[3] Hotamisligil GS. Inflammation, Metaflammation and Immunometabolic Disorders. Nature (2017) 542:177–85. doi: 10.1038/nature21363 28179656

[4] Rohm TV Meier DT Olefsky JM Donath MY. Inflammation in Obesity, Diabetes, and Related Disorders. Immunity (2022) 55:31–55. doi: 10.1016/j.immuni.2021.12.013 35021057 PMC8773457

[5] Roy P Orecchioni M Ley K. How the Immune System Shapes Atherosclerosis: Roles of Innate and Adaptive Immunity. Nat Rev Immunol (2022) 22:251–65. doi: 10.1038/s41577-021-00584-1 PMC10111155 34389841

[6] Caputa G Castoldi A Pearce EJ. Metabolic Adaptations of Tissue-Resident Immune Cells. Nat Immunol (2019) 20:793–801. doi: 10.1038/s41590-019-0407-0 31213715

[7] O'Neill LA Pearce EJ. Immunometabolism Governs Dendritic Cell and Macrophage Function. J Exp Med (2016) 213:15–23. doi: 10.1084/jem.20151570 26694970 PMC4710204

[8] Donath MY Dinarello CA Mandrup-Poulsen T. Targeting Innate Immune Mediators in Type 1 and Type 2 Diabetes. Nat Rev Immunol (2019) 19:734–46. doi: 10.1038/s41577-019-0213-9 31501536

[9] Palsson-McDermott EM O'Neill LAJ. Targeting Immunometabolism as an Anti-Inflammatory Strategy. Cell Res (2020) 30:300–14. doi: 10.1038/s41422-020-0291-z PMC7118080 32132672

[10] Guilliams M Bonnardel J Haest B Vanderborght B Wagner C Remmerie A. Spatial Proteogenomics Reveals Distinct and Evolutionarily Conserved Hepatic Macrophage Niches. Cell (2022) 185:379–96.e38. doi: 10.1016/j.cell.2021.12.018 35021063 PMC8809252

[11] Remmerie A Martens L Thone T Castoldi A Seurinck R Pavie B. Osteopontin Expression Identifies a Subset of Recruited Macrophages Distinct From Kupffer Cells in the Fatty Liver. Immunity (2020) 53:641–657.e14. doi: 10.1016/j.immuni.2020.08.004 32888418 PMC7501731

[12] Krenkel O Hundertmark J Abdallah AT Kohlhepp M Puengel T Roth T. Myeloid Cells in Liver and Bone Marrow Acquire a Functionally Distinct Inflammatory Phenotype During Obesity-Related Steatohepatitis. Gut (2020) 69:551–63. doi: 10.1136/gutjnl-2019-318382 31076404

[13] Jaitin DA Adlung L Thaiss CA Weiner A Li B Descamps H. Lipid-Associated Macrophages Control Metabolic Homeostasis in a Trem2-Dependent Manner. Cell (2019) 178:686–98.e14. doi: 10.1016/j.cell.2019.05.054 31257031 PMC7068689

[14] Hill DA Lim HW Kim YH Ho WY Foong YH Nelson VL. Distinct Macrophage Populations Direct Inflammatory Versus Physiological Changes in Adipose Tissue. Proc Natl Acad Sci USA (2018) 115:E5096–105. doi: 10.1073/pnas.1802611115 PMC5984532 29760084

[15] Emont MP Jacobs C Essene AL Pant D Tenen D Colleluori G. A Single-Cell Atlas of Human and Mouse White Adipose Tissue. Nature (2022) 603:926–33. doi: 10.1038/s41586-022-04518-2 PMC9504827 35296864

[16] van der Zande HJP Zawistowska-Deniziak A Guigas B. Immune Regulation of Metabolic Homeostasis by Helminths and Their Molecules. Trends Parasitol (2019) 35:795–808. doi: 10.1016/j.pt.2019.07.014 31492623

[17] Maizels RM Smits HH McSorley HJ. Modulation of Host Immunity by Helminths: The Expanding Repertoire of Parasite Effector Molecules. Immunity (2018) 49:801–18. doi: 10.1016/j.immuni.2018.10.016 PMC6269126 30462997

